# Relationship between perceived enjoyment, exercise commitment and behavioral intention among adolescents participating in “School Sport Club”

**DOI:** 10.3389/fmed.2023.1277494

**Published:** 2024-01-05

**Authors:** Kihong Joung, Wonjae Jeon, Goomyeung Kwon

**Affiliations:** ^1^Department of Physical Education, Kangnam University, Yongin, Republic of Korea; ^2^Department of Physical Education, Korea National University of Education, Cheongju, Republic of Korea; ^3^Department of Senior Sports Course, Daegu Haany University, Gyeongsan, Republic of Korea

**Keywords:** school sports club, perceived enjoyment, exercise commitment, behavioral intention, physical education, physical activity

## Abstract

This study aimed to examine ways to improve the quality of physical activity (PA) to address social problems related to obesity and being overweight among adolescents, through an educational approach. In this regard, the current study identified associations between factors that lead to sustained PA participation among middle school students participating in school sports club activities, and derived academic implications that can be incorporated into future school education programs. The researchers explored the relationship between perceived enjoyment, exercise commitment, and behavioral intention in middle school sports club participants. The subjects of the study were selected as middle school students who had participated in school sports clubs for more than 6 months. Using convenience sampling, 350 datasets were collected from middle school students living in metropolitan cities in South Korea. Finally, 336 datasets were used for the final analysis after eliminating 14 questionnaires that were judged to be incorrectly indicated or incomplete. Frequency analysis, exploratory factor analysis, and reliability verification (Cronbach’s α) were conducted. The findings were as follows: first, among the sub-factors of perceived enjoyment, the following were found to have a positive relationship with cognitive commitment: daily escape (*β* = 0.259), competitive victory (*β* = 0.228), interpersonal relationships (*β* = 0.204), and physical fitness (*β* = 0.119). Furthermore, among the sub-factors of perceived fun, physical health (*β* = 0.330), daily escape (*β* = 0.205), interpersonal relationships (*β* = 0.307), and competitive victory (*β* = 0.228) had positive relationships with behavioral commitment. Second, among the sub-factors of perceived enjoyment, physical health (*β* = 0.423), interpersonal relations (*β* = 0.139), and daily escape (*β* = 0.138) were found to have a positive association with behavioral intention. On the other hand, there was no significant relationship between competitive winning (*β* = 0.071) and behavioral intention. Third, behavioral commitment (*β* = 0.237) and cognitive commitment (*β* = 0.183) were confirmed to have a significant positive relationship with behavioral intention. These findings highlight that middle school students’ perceived enjoyment from participating in school sports clubs is a positive factor leading to increased immersion in sports activities and a sufficient basis for continuing sports activities. Furthermore, class organization, environmental factors, and appropriate instructional content for school sports club activities are essential for exercise commitment.

## 1 Introduction

Adolescence is the middle stage of growth that continues until adulthood. It is a period in which individuals focus on personality development and learn to adapt to society (1, 2). As such, physical activities performed in adolescence have been found to be effective in improving mental and physical health, and social, academic, and lifestyle skills (3, 4). However, according to various domestic and foreign studies (5–7) that analyzed the current status of physical activity in South Korean youth by age group, the recommended PA (60 min of high-intensity PA per day) achievement rate of male and female remained significantly low at 53 and 38.1%, respectively. In addition, the greater the intensity of exercise, the larger the difference in PA, and a lack of PA among adolescents leads to poor health (7). Essentially, adolescents’ energy intake exceeds their energy consumption; consequently, the probability of youths developing adult diseases is increasing (8), and the age of onset of adult diseases in adolescents is decreasing (9).

School physical education (PE) has attracted attention as youth health problems worsen in South Korea. This is because most teenagers spend a considerable amount of time in school and school PE targets health problems as its major educational goal (7). Most schools have professional manpower (e.g., P.E. teachers) and an efficient curriculum operation system that can help manage youth health problems (10). Nevertheless, this is not the case in South Korea. As mentioned above, most of South Korean adolescents spend most of their time at school, but the amount of PA time suitable for adolescence is far from sufficient. For example, as most schools focus on university entrance education, free PA time is not guaranteed except for regular PE classes (2 to 3 times a week, 45 to 50 min) (11). In particular, in South Korea, community PA facilities and programs for teenagers are scarce. Therefore, active efforts by school P.E programs are required to solve youth health problems (12).

PE systems in school are majorly responsible for physical activities in adolescence. However, there has been a growing perception that health promotion cannot be achieved by regular sports activities (P.E. classes) alone (9). The reason for this is that the number of P.E. classes in Korean schools is extremely insufficient compared with a majority of advanced countries (7). The United States Department of Health and Human Services raised the need for comprehensive school sports operations to promote PA throughout school life based on a daily exercise system (13). In fact, many countries have focused on providing sufficient PA for adolescents through programs such as the Comprehensive School-based PA Program (USA), Active School Flag (Ireland), Schools on the Move (Finland), and Moving Schools (Germany) (9). In South Korea, school sports club activities (operating as regular class activities once or twice a week) and after-school sports activities (operating as autonomous sports activities once or twice a week) have been operating under the school sports reinforcement policy since 2012 (14). Recently, the school sports club system policy has been implemented to achieve the effect of daily sports (sports activities five days a week) (15). Middle school students perceive school sports club activities as a time to act apart from their daily lives, to escape academic pressure, and to spend time with friends (16). For this reason, the Korean Ministry of Education has actively extended support for middle school students’ school sports club activities (17).

However, regardless of the educational value of continuous school sports club activities, these activities cannot last long unless enjoyment is guaranteed (18). From this perspective, the importance of enjoyment in participation and continuation of an activity is emphasized (19). In recent years, social problems such as bullying, school violence, obesity, being overweight, and sedentary school lifestyles among teenagers have become serious in South Korea (7). The Ministry of Education has implemented various educational initiatives to solve these problems; school sports club activities are at the center of these efforts (17). Continuous efforts and attention are needed to actively promote and encourage sports activities through school sports clubs in all schools. In particular, the focus should be on the fact that middle school students experience psychological aspects of fun and pleasure in most aspects of their daily lives. Enjoyment in sports activities is a positive emotional experience, involving passion, pleasure, and fun, obtained through physical activities (20). Perceived enjoyment affects factors such as challenges, overcoming challenges, and gaining confidence (21). These factors include the perception of competence and confidence through the improvement of sports skills, tension or excitement experienced in competitive situations, and formation of fraternity with peer groups (22–24). Based on these findings, it is necessary to develop a more focused program on enjoyment factors to further maximize the effectiveness of middle school students’ sports club activities (25).

The enjoyment of participating in sports leads to commitment to exercise or PA, which increases participation persistence (26). Exercise commitment can be defined as a physical and psychological state of desire or willingness to continue exercising and increase the desire to participate through positive thinking about exercise (27). Exercise commitment is used as an indicator of the degree of internal motivation, and refers to the state of immersion in a specific activity (28). Exercise commitment not only leads to positive results but can also be strengthened through satisfaction with the results (29). Moreover, exercise commitment is presented as a major factor in explaining outcome expectations (the more value and importance the outcome is recognized as having, the more likely it is to be acted upon) (30). That is, exercise commitment may be an important factor in sustaining sports participation, and is related to the exercise outcome expectation that induces more active participation in sports (31). Eventually, enjoyment increases exercise commitment, which may be a significant factor mediating continuous participation in exercise and sports (32).

Behavioral intention refers to future planned behavior as a direct factor in determining behavior in a decision-making model (33). It is defined as an individual’s will or belief that manifests as satisfaction or dissatisfaction with a particular activity (34). Since the Behavioral Intention Model was proposed, behavioral intent has been used in many studies as a decision variable. Fishbein and Ajzen (35) explained that individual behavior is directly affected by the degree of intention to perform. In general, because people behave according to certain intentions, behavioral intention is a vital indicator of an individual’s behavior (36). Furthermore, when an attitude toward a specific object is formed, an individual has the will and belief to perform a specific future behavior; therefore, behavioral intention can be utilized as an important predictor of behavior to achieve an individual’s goals (37, 38).

**Figure 1:**
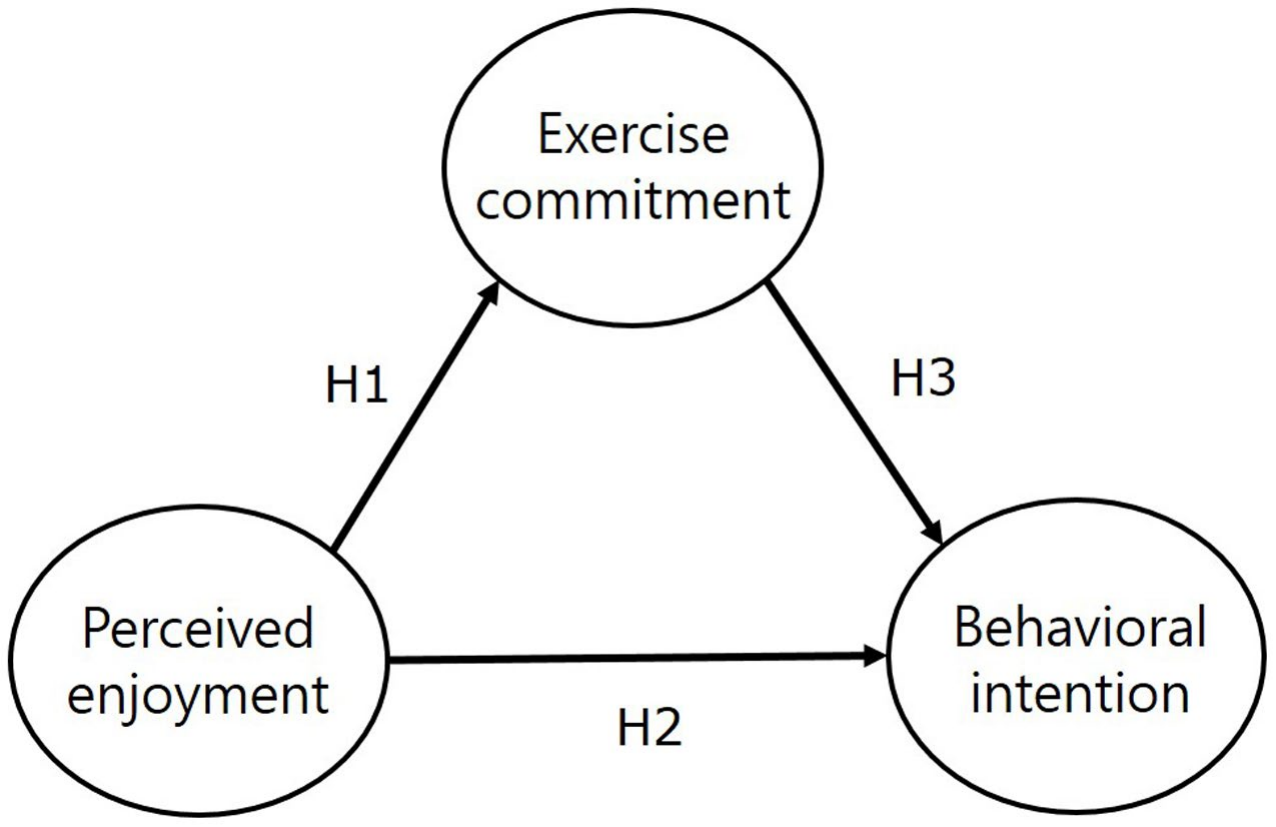
Research model.

In previous studies, a positive influence of perceived enjoyment on exercise commitment was reported for water leisure activity participants, college students participating in windsurfing classes, and middle school P.E. class participants (32, 39, 40). An influential relationship was also found between perceived enjoyment and behavioral intention in college winter-class participants and middle school dance-class participants (41, 42). Furthermore, perceived enjoyment had a significant relationship on exercise commitment and behavioral intention in baseball club members and bowling participants (43, 44). Given that perceived enjoyment of PA participation is strongly related to exercise commitment and has a positive association on persistent behavioral intention, it is necessary to prove the association between these three factors in the context of school sports clubs involving middle school students. In particular, this study highlights perceived enjoyment as an important factor in maintaining PA among adolescents and may have useful implications for future program organization and management of school sports club class. To achieve the research purpose, a conceptual model of the hypotheses was developed (Figure 1).

The hypotheses corresponding to each path in the conceptual model are:

*H1*: Perceived enjoyment of school sports club activities will have a positive association with exercise commitment.

*H2*: Perceived enjoyment of school sports club activities will have a positive association with behavioral intention.

*H3*: Exercise commitment in school sports club activities will have a positive association with behavioral intentions.

## 2 Methods

Based on previous studies, we found that perceived enjoyment of PA participation is closely related to exercise commitment and positively associated with continued behavioral intention. Furthermore, this study aims to investigate the relationship between perceived enjoyment, exercise commitment, and behavioral intention in order to promote voluntary and continuous participation in school sports clubs among middle school students. The detailed research model is as follows (Figure 1).

### 2.1 Participants

Middle schools in Seoul and Gyeonggi-do, South Korea, were selected as the target population. For sampling, 350 participants who had participated in school sports clubs for more than six months were selected using convenience sampling. The researchers visited middle schools in person to place advertisements for research participation and conducted a survey only with students who expressed their intention to voluntarily respond.

### 2.2 Measurements of key variables

A structured questionnaire based on previous studies and theories was used for assessment. It comprised 30 questions, including 2 items on demographic characteristics, 12 on perceived enjoyment, 12 on exercise commitment, and 4 on behavioral intention. Specifically, perceived enjoyment was measured by modifying the items used by Kim and Seong (45) and Yoon et al. (46). The perceived enjoyment scale consisted of 12 questions with four sub-factors: daily escape (3 questions), physical health (3 questions), interpersonal relations (3 questions), and competitive victory (3 questions). The exercise commitment scale, developed by Scanlan et al. (27) and translated into Korean by Jeong (28), was modified and measured according to the study purpose. It consisted of two factors: cognitive commitment (8 questions) and behavioral commitment (4 questions). Behavioral intention was assessed by adapting the questions in Zeithaml et al. (37). There were four questions (willingness to rejoin, talk positively, recommendations for activities and personal time and expense investment).

### 2.3 Procedure and statistical analysis

The researchers visited the schools and asked P.E. teachers for their cooperation in the survey. Furthermore, consent forms for study participation were distributed to the students to obtain their parents’ consent. After explaining the purpose and method for completing the questionnaire, the survey was conducted using the self-evaluation technique. If a face-to-face survey was not possible because of COVID-19, the purpose of the study and the survey method were delivered through the ZOOM program, and the questionnaire was sent and collected via e-mail (electronic signature). The survey was conducted once and took approximately 10–15 min to complete. Finally, before the survey was conducted, voluntary participation in the research was announced, and students who were unwilling to participate were excluded from the survey. Among the sampled survey data, 336 datasets were used for the final analysis after eliminating 14 questionnaires that were judged to be incorrectly indicated or incomplete. The questionnaires were removed by experts (statistical analysis experts and two doctors in the sociology of sports) other than the researchers.

Responses to the measurement tools were recorded on a five-point Likert scale (1 = strongly disagree to 5 = strongly agree). To examine the validity of the structured questionnaire, content validity was verified based on the advice of two professors majoring in sports sociology and two doctors. Exploratory factor analysis (EFA) using the maximum likelihood method and reliability analysis using Cronbach’s α coefficient were conducted to confirm the validity and reliability, respectively, of the measurement tools. The outcomes of the EFA are presented in Table 1. In particular, behavioral intention was a single factor, and the Cronbach’s α value was 0.887.

**Table 1:** Exploratory factor analysis (EFA) and reliability of latent variables.

	Classification	Daily escape	Physical health	Interpersonal relations	Competitive victory
Perceived enjoyment	Stress	**0.884**	0.022	0.034	0.032
	Freedom	**0.868**	0.062	0.007	0.045
	Pleasure	**0.863**	0.018	0.011	0.052
	Physical health	0.015	**0.912**	0.033	0.030
	Mental health	0.139	**0.889**	0.046	0.060
	Physical strength	0.120	**0.828**	0.040	0.018
	Making friends	0.014	0.032	**0.916**	0.040
	Getting close to friends	0.031	0.032	**0.877**	0.062
	Getting along well with friends	0.050	0.032	**0.825**	0.017
	Fun of competition	0.053	0.058	0.046	**0.894**
	Improvement of athletic ability	0.135	0.030	0.1137	**0.863**
	Fun of winning or losing	0.243	0.019	0.035	**0.717**
	Eigen value	2.398	2.320	2.312	2.074
	Common variance (%)	19.986	19.336	19.266	17.282
	Total variance (%)	19.986	39.322	58.588	75.870
	Reliability	0.850	0.846	0.847	0.774

	Classification	Cognitive commitment	Behavior commitment
Exercise commitment	Fun to think about	0.822	0.135
	Wait for participation	0.789	0.246
	Precious in life	0.764	0.137
	Continue to participate	0.755	0.247
	Feeling happy about participating	0.752	0.164
	Do more if I have time	0.694	0.222
	Gather information about skill	0.143	0.816
	I think I’m into exercise	0.228	0.745
	Imagining a great exercise performance	0.231	0.716
	View exercise media in priority	0.142	0.714
	Eigen value	3.646	2.479
	Common variance (%)	36.463	24.787
	Total variance (%)	36.463	61.249
	Reliability	0.877	0.770

1. K.M.O = 0.789, Bartlett’s, *p* < 0.001. 2. K.M.O = 0.868, Bartlett’s, *p* < 0.001. Exploratory factor analysis shows the results of grouping factors together.

### 2.4 Statistical analysis

The collected data were processed as follows: first, a frequency analysis was conducted. Second, confirmatory factor analysis and reliability verification (Cronbach’s α) were conducted to verify the validity of the survey tool. Third, correlation and standard multiple regression analyses were performed. All analyses were conducted using the SPSS 21.0 program. The probability of statistical significance was set at 0.05.

## 3 Results

### 3.1 Characteristics of participants

The specific characteristics of the participants in this study were as follows. Of the 336 participants, 192 were male (57.1%) and 144 were female (42.9%). In terms of grade, 97 students were in first grade (28.9%), 113 students were in second grade (33.6%) and 126 students were in third grade (37.5%).

### 3.2 Correlation analysis among study variables

As shown in Table 2, the correlation between each factor determined the satisfaction of discriminant validity between each factor for those with a single dimensionality. There was a partially significant correlation (r) between the relevant variables (Table 2). Because the values of all correlation coefficients did not exceed 0.80, discrimination was obtained based on the criteria of Kline (47). Additionally, all variables were lower than 0.80, which is the criterion for multicollinearity between independent variables, indicating that there was no multicollinearity problem (48).

**Table 2:** Correlation analysis among variables.

Variables	1	2	3	4	5	6	7
Daily Escape	1						
Physical Health	0.026	1					
Interpersonal Relations	0.160**	0.123*	1				
Competitive Victory	0.174**	0.104	0.124*	1			
Cognitive Commitment	0.122*	0.172**	0.258***	0.220***	1		
Behavior Commitment	0.324***	0.302***	0.193***	0.111*	0.668***	1	
Behavioral Intention	0.418***	0.138**	0.149**	0.065	0.128*	0.198***	1

*p < 0.05, **p < 0.01, ***p < 0.001. 1. Daily escape, 2. Physical health, 3. Interpersonal relations, 4. Competitive victory, 5. Cognitive commitment, 6. Behavior commitment, 7. Behavioral intention.

### 3.3 Effect of perceived enjoyment on exercise commitment

Table 3 shows the results of multiple regression analysis to verify the influence of perceived enjoyment on exercise commitment. Perceived enjoyment had a significant association on cognitive commitment (*p* < 0.001), and the explanatory power of the regression model was approximately 26.7% (R^2^_adj_ = 25.7%). The Durbin–Watson (DW) statistic was 2.090, showing a value close to 2, which was evaluated as no problem in the independence assumption of the residuals. The Variance Inflation Factor (VIF) was also found to be less than 10, indicating that there was no multicollinearity problem. After verifying the significance of the regression coefficients, daily escape (*β* = 0.259, *p* < 0.001), competitive victory (*β* = 0.228, *p* < 0.001), interpersonal relationships (*β* = 0.204, *p* < 0.001), and physical fitness (*β* = 0.119, *p* < 0.05) were confirmed to have significant positive relationships with cognitive commitment. In other words, the higher the daily escape, competitive victory, interpersonal relationships, and physical health, the higher the cognitive commitment.

**Table 3:** Effect of perceived enjoyment on exercise commitment.

	Section	*β*	t		Section	*β*	t	VIF
Cognitive commitment	Daily escape	0.259	5.156***	Behavior commitment	Daily escape	0.200	3.9281***	1.001
	Physical health	0.119	2.358*		Physical health	0.330	6.494***	1.004
	Interpersonal relations	0.204	4.043***		Interpersonal relations	0.142	2.470*	1.011
	Competitive victory	0.228	4.513***		Competitive victory	0.130	2.201*	1.014
*F* = 16.545 (*p* < 0.001), R^2^ = 0.267, R^2^_adj_ = 0.257, DW = 2.090				*F* = 14.291 (*p* < 0.001), R^2^ = 0.147, R^2^_adj_ = 0.137, DW = 2.151				

*p < 0.05, **p < 0.01, ***p < 0.001.

The regression model for the relationship of perceived enjoyment on behavioral commitment was statistically significant (*p* < 0.001), and the explanatory power of the model was approximately 14.7% (R^2^_adj_ = 13.7%). The DW statistic was 2.151, showing a value close to 2, which was evaluated as no problem in the independence of the residuals. On verifying the significance of the regression coefficients, physical health (*β* = 0.330, *p* < 0.001), daily escape (*β* = 0.205, *p* < 0.001), interpersonal relationships (*β* = 0.307, *p* < 0.05), and competitive victory (*β* = 0.228, *p* < 0.05) were confirmed to have significant positive associations with behavioral commitment.

### 3.4 Effect of perceived enjoyment on behavioral intention

Table 4 presents the results of the multiple regression analysis to verify the influence of perceived enjoyment on behavioral intention. Perceived enjoyment showed a statistically significant relationship on behavioral intention (*p* < 0.001), and the explanatory power of the regression model was approximately 22.1% (R^2^_adj_ = 21.2%). The DW statistic was 1.926, which was approximately 2 and evaluated as no problem in the independence assumption of the residuals. The VIF was less than 10, indicating that there was no multicollinearity. On verifying the significance of the regression coefficients, physical health (*β* = 0.423, *p* < 0.001), interpersonal relations (*β* = 0.139, *p* < 0.01), and daily escape (*β* = 0.138, *p* < 0.01) were confirmed to have significant positive relationships with behavioral intention. However, there was no significant relationship between competitive victory (*β* = 0.071) and behavioral intention.

**Table 4:** Effect of perceived enjoyment on behavioral intention.

	Section	*β*	*t*	VIF
Behavioral intention	Daily escape	0.138	2.844**	1.001
	Physical health	0.423	8.712***	1.004
	Interpersonal relations	0.139	2.849**	1.011
	Competitive victory	0.071	1.460	1.014

R^2^ = 0.221, R^2^_adj_ = 0.212, DW = 1.926. *p < 0.05, **p < 0.01, ***p < 0.001.

### 3.5 Effect of exercise commitment on behavioral intention

Table 5 shows the results of the multiple regression analysis between exercise commitment and behavioral intention. Exercise commitment had a statistically predictive association on behavioral intention (*p* < 0.001), and the explanatory power of the regression model was 14.52% (R^2^_adj_ = 13.9%). The DW statistic was 2.014, which was close to 2, determining that there was no problem in the independence of the residuals. The VIF was less than 10, indicating that there was no multicollinearity. As a result of verifying the significance of the regression coefficients, behavioral commitment (*β* = 0.237, *p* < 0.001) and cognitive commitment (*β* = 0.183, *p* < 0.05) were confirmed to have a significant positive relationship with behavioral intention.

**Table 5:** Effect of exercise commitment on behavioral intention.

	Section	*β*	*t*	VIF
Behavioral intention	Cognitive commitment	1.83	1.376*	1.281
	Behavior commitment	2.37	3.912***	1.281

R^2^ = 0.145, R^2^_adj_ = 0.139, DW = 2.014. *p < 0.05, **p < 0.01, ***p < 0.001.

## 4 Discussion

According to Trost (49), PA during adolescence has been shown to have positive effects on health development and future health maintenance in adulthood, so adolescent health policies and education should focus on PA. Specifically, sports club activities in which adolescents can freely participate at school are among the most important educational activities promoting PA (16). Hence, it is crucial to establish a theoretical basis for increasing adolescent participation in sports club activities and maximizing PA. Based on this theoretical perspective, this study attempted to investigate the relationship between perceived enjoyment, exercise commitment, and behavioral intention to induce voluntary and continuous participation of middle school students in school sports clubs. Participating in various sports activities in school plays an important role in satisfying the desire for PA, decreasing obesity, improving physical strength, preventing school violence, and developing personality (15–17).

First, considering the present results, students participating in middle school sports clubs experienced enjoyment through physical health, daily escape, interpersonal relations, and competitive victory. Perceived enjoyment had a significant association on cognitive and behavioral commitment. To be more specific, among the sub-factors of perceived enjoyment, the following were found to have a positive relationship with cognitive commitment: daily escape (*β* = 0.259), competitive victory (*β* = 0.228), interpersonal relationships (*β* = 0.204), and physical fitness (*β* = 0.119). Furthermore, among the sub-factors of perceived fun, physical health (*β* = 0.330), daily escape (*β* = 0.205), interpersonal relationships (*β* = 0.307), and competitive victory (*β* = 0.228) had positive relationships with behavioral commitment. Ultimately, middle school students’ participation in school sports clubs means that they are engaged in healthy physical activities that can protect their physical and mental health. In addition, students can relieve stress and experience escape and freedom from daily life chores by voluntarily participating in sports, forming new interpersonal relations, and experiencing a variety of emotions in competitive victory and defeat (40). This perceived enjoyment may be a significant means of experiencing happiness in life by positively influencing cognitive and behavioral commitment to exercise and sports (39), and continuous sports participation in the future (50). The competitive element and positive perception of the physical aspect acquired through school sports club activities may serve as driving forces for further engagement in sports activities. In addition, positive interpersonal relationships formed through sports activities and special experiences in daily life or school are important factors that lead to a greater focus on sports activities (51). Thus, it can be inferred that, among the current study participants, school sports club activities significantly influenced exercise commitment, physical and psychological health, social cultivation, and a sense of achievement. If middle school students actively participate in school sports club activities and experience enjoyment during the process, exercise commitment is promoted (52). Thus, it is possible to confirm a scientific basis for the effect of perceived enjoyment on exercise commitment in school sports clubs activities in which the students in this study participated.

Second, in terms of analyzing the relationship between perceived enjoyment and behavioral intention of participants in middle school sports clubs, the factors of daily escape, interpersonal relations, and physical health significantly influenced behavioral intention. Among the sub-factors of perceived enjoyment, physical health (*β* = 0.423), interpersonal relations (*β* = 0.139), and daily escape (*β* = 0.138) were found to have a positive association with behavioral intention. On the other hand, there was no significant relationship between competitive winning (*β* = 0.071) and behavioral intention. More specifically, middle school students’ participation in sports clubs was one of the most important ways to feel free from daily routines and relieve stress. In addition, the other factors in the perceived enjoyment, which is associated with the development of physical and mental health and the formation of peer relationships, is an important variable that positively affects behavioral intention. Previous studies have found a significant relationship between perceived enjoyment and behavioral intention obtained from marine leisure experiences. The academic implication is that enjoyment from participation in scuba diving, a marine recreational activity, can lead to positive behavioral intentions (53). Yoon et al. (41) noted that the enjoyment factors involved in college students’ participation in skipping and snowboarding classes had a positive association on their intention to continue exercising, and the higher the level of enjoyment, the higher their behavioral intention. Furthermore, Park et al. (54) emphasized that university students’ perceived enjoyment from participation in liberal arts golf classes had a positive influence on their exercise adherence intention. Hence, perceived enjoyment obtained from participating in various sports activities positively affects behavioral intention. In fact, if one does not experience enjoyment when participating in a sports activity, one is very likely to drop out of it; the need for perceived enjoyment is an important factor in increasing behavioral intention (26). Consequently, the positive relationship between perceived enjoyment and behavioral intention found in this study provides an important basis for sustained participation in school sports clubs among middle school students (42). However, the factors of competition and victory experienced by the participants had no effect on behavioral intention. According to Scanlan & Simons (55), sports enjoyment refers to intrinsic rather than extrinsic feelings such as fun, liking, and enjoyment, and these positive emotional responses influence continued sports participation. In addition, Wankel et al. (24) stated that intrinsic rewards such as enjoyment, fun, and having a good time are the most important factors in sports participation. Therefore, administrators of school sports clubs (e.g., P.E. teachers) need to pay more attention to enjoyment factors in planning sports activities (56).

Third, as a result of verifying the significance of the regression coefficients, behavioral commitment (*β* = 0.237) and cognitive commitment (*β* = 0.183) were confirmed to have a significant positive relationship with behavioral intention. In other words, exercise commitment gained through school sports club activities may be an important factor influencing students’ behavioral intentions. Various studies have examined this relationship. Min et al. (57) stressed that exercise commitment perceived by triathlon club members had a positive effect on exercise continuity behavior. Moreover, exercise commitment in social baseball had a positive relationship on exercise satisfaction and exercise continuity behavioral intention (43). Yoon and Lee (41) showed that exercise commitment perceived by college students participating in skiing and snowboarding classes had a significant effect on continuous exercise behavior. These results suggest that increasing exercise commitment is important in school sports club activities. In particular, the findings regarding a positive association of exercise commitment on behavioral intention of students participating in after-school physical education classes (58) support the results of this study. As exercise commitment is an important factor in the continued intention to participate in sports activities (59), the exercise commitment experienced by students in school sports clubs may be one of the most important factors. Therefore, reasonable activity operation management that can increase students’ desire and willingness to continue participating in school sports clubs is essential. Additionally, environmental factors that can induce students to exercise should be considered. For example, facility conditions and class atmosphere are important. Most importantly, the club operator, for example, P.E. teachers, should consider which sports event to choose. In other words, sports club activities should be designed primarily for sports that induce students’ commitment to exercising. This increases the chances of creating an educational environment that encourages students to participate in sports clubs, from a long-term perspective.

To promote student engagement in PA, Mohammadi et al. (60) suggested that schools increase the frequency and duration of P.E. classes. Furthermore, in order to expand the physical activity program of adolescents, the concept of physical literacy (PL) needs to be dealt with in school physical education (61). In particular, PL can expand physical activity in relation to adolescents’ holistic development (62). Therefore, it will be necessary to develop specific programs and manuals that can teach PL through sports club activities. In addition, PE teacher education for physical activity education based on PL should be accompanied. In South Korea’s physical education curriculum, the number of hours of P.E. classes per week is set by law. Therefore, institutional changes are needed to allow students to participate in sports club activities in their spare time. In other words, there is a need to find ways to increase the frequency and duration of PA in schools. Moreover, the results suggest that sports club activity programs should focus more on students’ perceived enjoyment. Based on the results of this study, sports club activities that can increase adolescents’ exercise commitment need to be further encouraged, and it is considered necessary to develop programs that can include PL. Eventually, through these programs, it will be possible to increase the behavioral intention of adolescents’ sports club activities, and further lead to long-term participation.

On the other hand, this study has the following limitations. First, this study focused on continuous PA, which limits its ability to address a range of youth social problems, including overweight and obesity. Second, as a limitation of this study, Korean school sports club events are operated in various sports by schools in each region, so it was difficult to present specific programs for each sport event in this study. Therefore, future research should be conducted on the development of specific sports club events and detailed programs to be introduced by individual physical education teachers according to students’ voluntary participation and needs.

## 5 Conclusion

The current study attempted to derive the academic implications of middle school students’ continuous participation in school sports club activities. The researchers explored the relationship between perceived enjoyment, exercise commitment, and behavioral intention of middle school sports club participants in a metropolitan city in South Korea.

First, it was found that competitive factors and the positive perception of fitness aspects acquired by students in school sports club activities can be positive factors for further immersion in sports activities. In particular, the positive experience of interpersonal relationships formed in sports activities can be an opportunity to focus more on physical activities. Thus, the hypothesis that perceived enjoyment from active participation in school sports club activities promotes exercise commitment was supported. Second, perceived enjoyment gained from participating in school sports clubs became a sufficient basis for the continuity of physical activity. Accordingly, it is suggested that school sports club activities be planned and conducted with more emphasis on the enjoyment that comes from the activity itself, rather than the importance of transferring classroom knowledge. Third, exercise commitment had a positive predictive association on behavioral intention. Previous studies have shown that class organization, environmental factors, and appropriate instructional content are essential for increasing exercise engagement in school sports club activities (15, 16, 25). Therefore, it is necessary to create an educational environment in which students can become immersed in physical activities to induce behavioral intentions.

Based on the above findings and discussion, suggestions for follow-up research are outlined. First, there is a need for research on factors other than enjoyment that can attract students’ interest and increase their participation rate in the planning and operation of middle school sports clubs. Second, additional qualitative research should be conducted to understand the students’ specific perceptions of participating in middle school sports clubs. Third, based on the findings of this study, it is necessary to apply the Delphi technique with a group of experts to develop specific PA programs that can be implemented in schools in the future.

## Data availability statement

The original contributions presented in the study are included in the article/supplementary material, further inquiries can be directed to the corresponding authors.

## Ethics statement

The studies involving humans were approved by Kangnam University Institutional Review Board (NO. KNU-HR2109001). The studies were conducted in accordance with the local legislation and institutional requirements. Written informed consent for participation in this study was provided by the participants' legal guardians/next of kin. Written informed consent was obtained from the minor(s)' legal guardian/next of kin for the publication of any potentially identifiable images or data included in this article.

## Author contributions

KJ: Data curation, Formal analysis, Investigation, Supervision, Writing – original draft. WJ: Conceptualization, Data curation, Software, Supervision, Writing – original draft. GK: Methodology, Project administration, Resources, Supervision, Validation, Visualization, Writing – review & editing.
